# Multidrug-resistant among Non-Fermenting Gram-negative Bacteria Isolated in the Department of Microbiology of a Tertiary Care Centre

**DOI:** 10.31729/jnma.8330

**Published:** 2023-11-30

**Authors:** Sabita Bhatta, Manoj Pradhan, Raina Chaudhary

**Affiliations:** 1 Department of Microbiology, Nepalese Army Institute of Health Sciences, Sanobharyang, Kathmandu, Nepal

**Keywords:** *Acinetobacter baumannii*, *antibiotic*, *Pseudomonas aeruginosa*, *prevalence*

## Abstract

**Introduction:**

Infection caused by Non-fermenting Gram-negative bacteria (NFGNB) like *Pseudomonas aeruginosa* and *Acinetobacter baumannii* leads to life-threatening conditions. These bacteria are often multidrug-resistant which leads to limited therapeutic options leading to treatment failure. Little information is available regarding the prevalence and resistance pattern of such bacteria in our country. The aim of the study was to find out the prevalence of multidrug-resistant among nonfermenting Gram-negative bacteria isolated in the Department of Microbiology of a tertiary care centre.

**Methods:**

A descriptive cross-sectional study was conducted in the Department of Microbiology of a tertiary care centre from 1 September 2021 to 30 August 2022 after obtaining ethical approval from the Institutional Review Committee. All samples received in the Microbiology laboratory for diagnostic purposes were included. A convenience sampling method was used. The point estimated was calculated at a 95% Confidence Interval.

**Results:**

Among 412 non-fermenting Gram-negative bacteria, multidrug resistance was observed in 373 (90.53%) (87.70-93.36, 95% Confidence Interval) isolates. Among 373 isolates, *Acinetobacter baumannii* was 253 (67.83%) and *Pseudomonas aeruginosa* was 120 (32.17%).

**Conclusions:**

The prevalence of multidrug-resistant non-fermenting Gram-negative bacteria was found to be higher than in the study conducted in similar settings.

## INTRODUCTION

Non-fermenting Gram-negative bacteria (NFGNB) are a diverse group of aerobic, non-spore-forming bacilli that do not utilize glucose as a source of energy or utilize it oxidatively.^1^ These are ubiquitous in nature and are found in ventilators, humidifiers and fomites in the hospital environment.^2^ Infections by NFGNB approximately account for 15% of total Gram-negative bacterial infections that are encountered in hospitals.^3^

NFGNBs are often multidrug-resistant (MDR) due to their intrinsic or acquired mechanism of antibiotic resistance.^4^ MDR organisms are increasing rampantly due to poor regulatory policies for the use of antibiotics thus leading to limited therapeutic options. Therefore, it is important that Microbiologists should monitor the prevalence of MDR isolates with their resistance pattern for better management of the patient.

The aim of the study was to find out the prevalence of multidrug-resistant among non-fermenting Gramnegative bacteria isolated in the Department of Microbiology of a tertiary care centre.

## METHODS

A descriptive cross-sectional study was conducted in the Department of Microbiology in Nepalese Army Institiute of Health Sciences, Sanobharyang, Kathmandu, Nepal of a tertiary care centre from 1 September 2021 to 30 August 2022 after obtaining ethical approval from the Institutional Review Committee (Reference number: 452/20). Among all samples (urine, pus, wound swabs, sputum, tracheal aspirate and sterile body fluids) received in the Microbiology laboratory for diagnostic purposes from which isolates containing *Acinetobacter baumannii* and *Pseudomonas aeruginosa* were included. Duplicate samples were excluded from the study. Convenience sampling method was used. The sample size was calculated using the following formula:


$$\begin{array}{ll} \text{n} & ={\text{Z}}^{2}\times \frac{\text{p}\times \text{q}}{{\text{e}}^{\text{2}}}\\ & =1.96^{2}\times \frac{0.50\times 0.50}{0.05^{2}}\\ & =384 \end{array}$$

Where,

n = minimum required sample size
Z = 1.96 at 95% Confidence Interval (CI)
p = prevalence taken as 50% for maximum sample calculation
q = 1-p
e = margin of error, 5%

The calculated sample size was 384. However, 412 isolates were taken for the study.

All these samples were processed as per standard guidelines mentioned in Clinical Laboratory Standard Institute (CLSI).^5^ Urine samples were inoculated in Cysteine Lactose Electrolyte Deficient (CLED) media while other samples were inoculated in blood agar, MacConkey agar and chocolate agar. Quality control of laboratory equipment, reagents and media was carried out regularly. Mueller Hinton Agar (MHA) and antibiotic disc were checked for their lot number, manufacture and expiry date and proper storage. For standardization of the Kirby-Bauer test and for performance testing of antibiotics and MHA, the control strain of *Escherichia coli* (ATCC 25922) and *Pseudomonas aeruginosa* (ATCC 27853) were tested primarily. Quality of sensitivity tests was maintained by maintaining the thickness of MHA at 4 mm and the PH at 7.2-7.4. MDR means an acquired non-susceptibility to at least one agent in three or more antimicrobial categories.^6^ Cefotaxime and ceftriazone are not recommended for use against *Pseudomonas aeruginosa* and ofloxacin aztreonam and levofloxacin are not recommended for use against *Acinetobacter baumannii*.^5^

Data was entered in Microsoft Excel 2016 and analysis was done using IBM SPSS Statistics version 18.0. The point estimate was calculated at a 95% CI.

## RESULTS

Among 412 NFGNB, MDR was observed in 373 (90.53%) (87.70-93.36, 95% CI) isolates. *Acinetobacter baumannii* was 253 (67.83%) and *Pseudomonas aeruginosa* was 120 (32.17%). MDR isolates were much higher in sputum 163 (59.49%) followed by wound swab/pus 141 (51.46%) (Table 1).

**Table 1:** Distribution of MDR isolates in a sample (n = 373).

Samples	n (%)
Sputum	163 (43.70)
Wound swab/pus	141 (37.80)
Tracheal aspirate	56 (15.01)
Urine	11 (2.95)
Sterile body fluid	2 (0.54)

Among MDR isolates, drugs were tested for susceptibility in which the piperacillin+tazobactam group of drugs were found to be less resistant that is 46 (16.79%) and 214 (78.10%) for both the organisms *Pseudomonas aeruginosa* and *Acinetobacter baumannii* (Table 2).

**Table 2:** Drug-resistant *Pseudomonas aeruginosa* and *Acinetobacter baumannii* to different classes of drugs (n= 373).

Drugs	Pseudomonas aeruginosa	Acinetobacter baumannii
Piperacillin	75 (62.5)	237 (86.50)
Piperacillin + tazobactum	46 (16.79)	214 (78.10)
Ceftazidime	105 (38.32)	243 (88.69)
Cefipime	90 (32.85)	245 (89.42)
Amikacin	70 (25.55)	248 (90.51)
Ciprofloxacin	109 (39.78)	245 (89.42)
Imipenem	72 (26.28)	217 (79.20)
Meropenem	59 (21.53)	217 (79.20)
Cefotaxime	-	248 (90.51)
Ceftriazone	-	240 (87.59)
Ofloxacin	98 (35.77)	-
Aztreonam	59 (21.53)	-
Levofloxacin	67 (24.45)	-

## DISCUSSION

Among NFGNB, MDR was observed in 90.53%. The previous study showed a prevalence of MDR was found to be 91% and 73.3% for *Acinetobacter baumannii* and *Pseudomonas aeruginosa* respectively.^7,9^ A major difference in prevalence rate may be due to consideration of sample size, the inclusion of *Pseudomonas aeruginosa* and *Acinetobacter baumannii* only among NFGNB.

MDR isolates were much higher in sputum 163 (59.49%) followed by wound swab/pus 141 (51.46%) which was in concordance with other studies conducted in similar settings^7,9,10^ Among different drugs tested for susceptibility, piperacillin+tazobactam group of drugs were found to be less resistant that is 46 (16.79%) and 214 (78.10%) for both the organisms *Pseudomonas aeruginosa* and *Acinetobacter baumannii.* For *Acinetobacter baumannii,* resistance against imipenem and meropenem was 85.7% and piperacillin+tazobactam was (84.5%) which was almost in concordance with other studies while for *Pseudomonas aeruginosa*, for imipenem (60%), meropenem (49.1%) and piperacillin+tazobactam (38.33%) which was not in concordance with other studies.^7^

This is a single-centric study with a small sample size. The effectiveness of susceptible drugs and the outcome of the patients with NFGNB infections could not be monitored due to insufficient data. Genotypic mechanisms for resistance were not detected due to a lack of resources. Besides *Acinetobacter baumannii* and *Pseudomonas aeruginosa,* other non-fermenters were not included as we were unable to perform minimum inhibitory concentration (MIC) for antibiotic susceptibility testing as per the CLSI recommendation.

## CONCLUSIONS

The prevalence of MDR NFGNB was higher as compared to other studies done in similar settings. Reliable data regarding the resistance patterns of drugs on a regular basis can help formulate antibiotic policy and strengthen antibiotic stewardship programmes.
